# Recurrent Monophasic Wilms‘ Tumor in Pelvic Kidney – A Therapeutic Challenge

**Published:** 2015-09-01

**Authors:** Yogesh Kumar Sarin, Prince Raj

**Affiliations:** Department of Pediatric Surgery, Maulana Azad Medical College, and associated LokNayak Hospital, New Delhi

**Keywords:** Wilms' Tumor, Pelvic Kidney, Recurrences, Lymphadenopathy

## Abstract

Wilms’ tumor in the current era of multimodality treatment has promising outcome, but approximately 10-15% of the patients with favorable-histology, experience tumor progression or relapse. We hereby present an unusual case of repeated loco-regional recurrences in a patient with stage I intermediate-risk monophasic (epithelial variant) Wilms’ tumor (WT) of pelvic kidney requiring aggressive therapy over a decade and lay emphasis on the importance of initial completion of therapy and need for long term follow-up.

## CASE REPORT

A one-year-old boy presented with lower abdominal lump, fever, and pain abdomen for one month. On abdominal examination a well-defined mass in the left lower quadrant was palpable. Radiological investigations including ultrasonography (USG) and contrast enhanced computed tomography (CECT) revealed mass in left ectopic kidney with no renal or inferior vena cava (IVC) thrombus (Fig. 1). Left nephroureterectomy was done with para-aortic and para-caval lymph node sampling. IVC was palpated but no thrombus was found. Contralateral kidney was normal and there were no enlarged lymph nodes.

**Figure:**
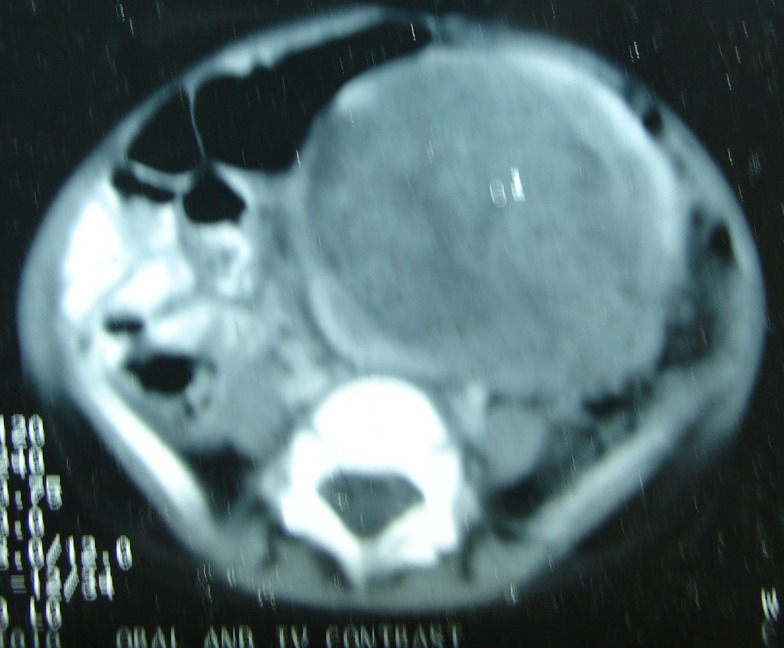
Figure 1:CECT scan showing 6x5cm mass in left ectopic kidney.

Histopathologic examination (HPE) showed WT with predominant tubule formation, minimal atypia and mitosis (stage I). Sampled lymph nodes did not show any evidence of tumor. The patient was referred elsewhere for further adjunct therapy, where there was a considerable delay before any adjunct therapy was initiated. Repeat CECT done at this stage in radiotherapy department showed thrombus in IVC and right common iliac vein with para-aortic and iliac lymphadenopathy (Fig. 2). After two cycles of VAC, family abandoned the therapy and was lost to follow-up. He returned with local relapse in retroperitoneal lymph nodes after 7 years. Partial calcified thrombus was present in sub-hepatic IVC on CECT. After 6 cycles of ifosfamide, cisplatin and etoposide (ICE Regime), retroperitoneal lymph node dissection (RPLND) was done; HPE showed no tumor.

**Figure:**
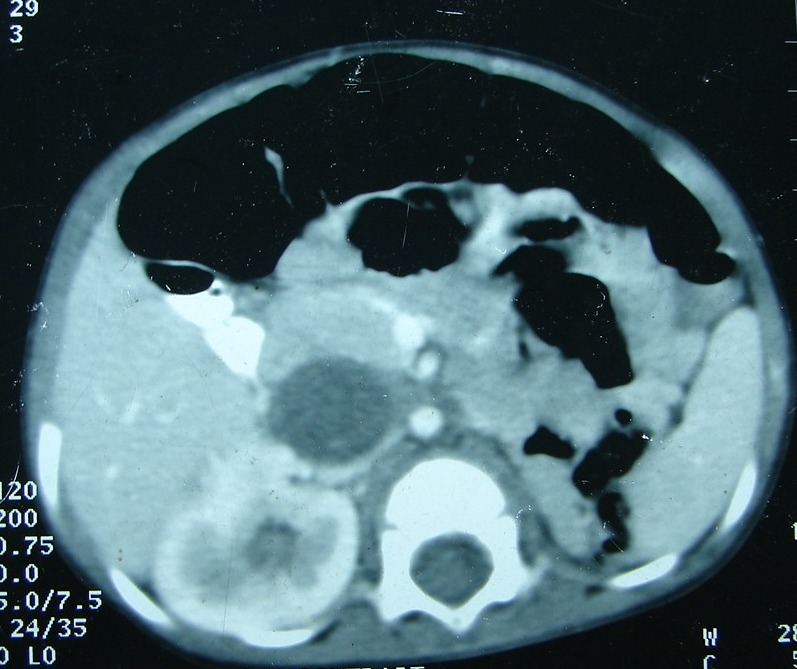
Figure 2:CECT scan showing IVC thrombus and lymphadenopathy.

Fourteen months later, he again presented with mass in abdomen. Repeat CECT showed partial calcified thrombus in sub-hepatic IVC, and recurrence in mesocolic lymph nodes that was completely excised. It was followed by 6 cycles of IE chemotherapy and 20 Gy whole abdominal irradiation. HPE was again reported as epithelial variant of WT. Four months later, he presented with third recurrence -as mass abdomen (15×8×7cm) in left hemi pelvis posterior to the bladder. CECT showed partial calcified thrombus in sub-hepatic IVC (Fig. 3). Tru-cut biopsy (not CT guided as the mass was palpable) again showed epithelial predominant WT. He received 6 cycles of paclitaxel based salvage chemotherapy followed by excision of the retro-vesical tumor and boost radiotherapy of 10.8 Gy to the pelvis. Six months later, he developed biopsy proven radiation nephritis and developed acute renal failure for which periodic hemodialysis was started. He succumbed to renal failure after few weeks at the age of 14 year.

**Figure:**
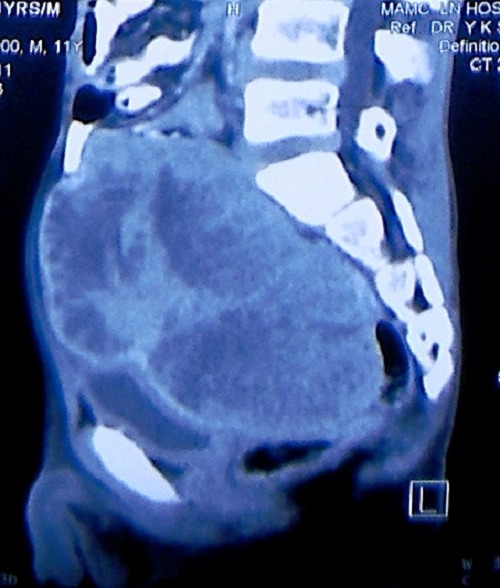
Figure 3:CECT scan showing mass lesion.

## DISCUSSION

WT is the most common renal malignancy in children, and its treatment is one of the great success stories in pediatric oncology with cure rates approaching around 90%. This improved outcome has been the result of development of biological and clinical prognostic factors, which have guided for risk directed therapy. However, even with current multimodality treatment, approximately 10-15% of patients with favorable-histology (FH) disease and 50% of patients with anaplastic tumors experience tumor progression or relapse. These relapses are quite heterogeneous and though some can be effectively treated with the standard chemotherapy regimens, other with poor prognostic factors have dismal outcome.[1]

Stage and histology are important prognostic factors and tumors with lower stage and favorable histology have better prognosis and lower relapse rate. Favorable prognostic factors after relapse include favorable histologic features, relapse occurring more than 12 months after the initial diagnosis, low stage (I or II) of primary disease, initial treatment with vincristine and dactinomycin only, few pulmonary nodules, and no previous irradiation of the tumor bed.[2] Index case complied with most of these favorable prognostic factors but it recurred each time. Though there was no palpable thrombus in IVC or in renal vein at the time of first surgery, but CECT done a month later revealed thrombus in IVC, which possibly could have been missed intra operatively. Surprisingly, even after early abandonment of adjunct therapy, he had event free survival for next seven years. This was followed by three loco-regional relapses in quick succession. Each time the tumor was excised completely and biopsy revealed favorable histology (epithelial predominant WT).

As there is no fixed protocol developed by either group regarding the chemotherapy drugs, these recurrences were managed by giving ICE regime in the first recurrence and IE regime in second recurrence. Cisplatin was omitted in the second recurrence in view of preventing nephrotoxicity and ototoxicity. In the third recurrence taxane based salvage chemotherapy was given. Such treatment policy was adopted as there are no readymade protocols for management of multiple recurrences. In view of multiple recurrences, it was decided to give abdominal radiation to the tumor bed after discussing in tumor board meeting.

This raises another important question as to what may be the cause of such behavior of this tumor. This emphasizes on the need to consider other biological markers, which could have role in such an adverse outcome. Tumor-specific LOH for both chromosomes 1p and 16q identifies a subset of FH Wilms’ tumor patients who have a significantly increased risk of relapse and death. LOH for these chromosomal regions can now be used as an independent prognostic factor together with disease stage to target intensity of treatment to risk of treatment failure.[3]

A lot more has to be done regarding the standard protocol, dosing and regimens based on the site of recurrence and risk stratification.[4] Currently novel agents like topotecan have shown significant anti tumor activity in cases of relapse WT when given for 5 days daily over two consecutive weeks.[5] Bevacizumab, an anti-vascular endothelial growth factor (VEGF) antibody inhibits the growth of Wilms’ tumor, prevent metastatic spread, and even induce tumor regression has also shown promise. The index case repeated recurrence was either due to unfavorable biological markers or the calcified tumor thrombus in IVC acting as a source for each relapse. Inert tumor cells in the calcified IVC thrombus had possibly caused the repeated loco-regional recurrences of a low-risk localized monophasic WT.

## Footnotes

**Source of Support:** Nil

**Conflict of Interest:** None declared

